# Anti-PD-1 Antibody Administration following Hip Fracture Surgery Reverses Immune Dysfunction and Decreases Susceptibility to Infection

**DOI:** 10.1155/2019/8492090

**Published:** 2019-04-03

**Authors:** Hao Zhang, Chuying Chen, Jiusheng He, Jianzheng Zhang, Zhi Liu, Tiansheng Sun

**Affiliations:** ^1^Department of Orthopedic Surgery, Beijing Shunyi District Hospital, Shunyi District, Guangming South Street No. 3, Beijing 101300, China; ^2^Department of Orthopedic Surgery, The Seventh Medical Center of PLA, Dongcheng District, Nanmencang No. 5, Beijing 100700, China

## Abstract

The aim of this investigation was to assess expression of programmed death-1 (PD-1) and inflammatory status after hip fracture surgery in aged mice and to evaluate the effect of anti-PD-1 antibody intervention. Male C57BL/6 mice aged 22-28 months underwent hip fracture and femoral intramedullary pinning or a sham procedure. Expression of PD-1 was measured on CD4^+^ and CD8^+^ T cells. Additionally, the effects of anti-PD-1 antibody on lymphocyte apoptosis, cytokine production, bacterial clearance, and survival were determined. Expression of PD-1 on T cells was upregulated in mice after hip fracture and surgery compared to sham controls. Administration of anti-PD-1 antibody prevented T lymphocyte apoptosis, increased IFN-*γ* production in splenocytes, and decreased systemic inflammation. Antibody blockade of PD-1 significantly decreased susceptibility to bacteria and improved survival rates of aged mice after hip fracture and surgery followed by the induction of *Pseudomonas aeruginosa* pneumonia. This study showed that hip fracture and surgical trauma cause significant increases in PD-1 expression in aged mice. Antibody blockade of PD-1 partially reverses T cell apoptosis, decreases the systemic inflammatory response and susceptibility to bacteria, and reduces mortality.

## 1. Introduction

As the global population ages, the incidence of hip fractures is increasing. Between 2002 and 2006, the incidence of hip fractures increased by 58% in women over 50 and by 49% in men over 50 in Beijing [1]. Although most hip fractures are treated with surgery, postoperative mortality can be as high as 26-30% within 1 year, and this rate has not significantly declined in the past 50 years [2, 3]. Hip fracture has become a major medical problem that seriously compromises patients' quality of life and the life expectancy of aged people.

Clinical evidence has shown that infectious complications, such as pulmonary infections, urinary infections, wound infections, and sepsis, are common after hip fracture surgery in elderly patients and seriously affect the prognosis [4, 5]. Severe trauma and surgery can elicit immunosuppression, which significantly inhibits biological processes such as recognition of antigens by immune cells, antigen presentation, T cell activation, and inflammatory cytokine secretion [6]. This decreases the body's ability to eliminate pathogenic bacteria and increases susceptibility to infection, which can lead to serious complications such as pulmonary infection, sepsis, and multiple organ failure [7]. Furthermore, as a result of weakened immune competence due to aging, older patients are more susceptible to immunosuppression caused by trauma and surgery and to infectious complications that may lead to death [8, 9]. Our previous study demonstrated that proximal femoral fracture and intramedullary nailing can lead to significant systemic inflammatory reactions, pathological damage to lung tissue, increased susceptibility to bacteria, and a decreased ability to eliminate bacteria in elderly rats [10]. However, there has been no direct evidence as to whether immunosuppression occurs in these animals.

Programmed death-1 (PD-1) is an important immunosuppressive molecule consisting of 288 amino acids and belonging to the CD28/CTLA-4 family [11]. It is expressed mainly on the surface of activated T cells, B cells, and the myeloid cell membrane, and its main function is to negatively regulate the immune response [12]. Numerous studies have shown that PD-1 plays a pivotal role in immunosuppression caused by tumors, chronic viral infections, and sepsis [13–15]. PD-1 also plays an important role in immune regulation in autoimmune diseases and organ transplant rejection [16, 17]. Furthermore, postoperative PD-1 expression is significantly higher in critically ill surgical patients and is closely related to postoperative immune dysfunction [18]. Because aging is associated with persistent chronic inflammation, negative feedback mechanisms related to immune regulation cause the expression of PD-1 to steadily increase with age [19]. Therefore, we speculated that inhibited immune functioning mediated by PD-1 may be an important cause of postoperative infectious complications in elderly hip fracture patients. The purpose of this study was to determine PD-1 expression patterns after proximal femoral fracture and intramedullary nailing in aged mice and to assess whether antibody blockade of PD-1 improves postoperative immune function and reduces lung infections and mortality.

## 2. Materials and Methods

### 2.1. Experimental Animals

A total of 114 male C57BL/6J mice aged 22-28 months were purchased from Haiwang Laboratory Animal Center (Beijing, China) and reared in animal-raising rooms (ordinary grade) at the Laboratory of Trauma, Orthopaedics Institute of The Seventh Medical Center of PLA. The room temperature was kept at 20-25°C with a relative humidity of 60-65% and natural lighting. Animals were given ad libitum access to food and water, and all animals were acclimated for 1 week prior to the experiment. Experiments were performed according to the guidelines for experimental animal care and use approved by The Seventh Medical Center of PLA.

### 2.2. Fracture Model and Surgery

Anterograde intramedullary nailing of a proximal femoral fracture was performed as described previously [10]. After anesthesia by intraperitoneal injection of xylazine (25 mg/kg) and ketamine (75 mg/kg), the mouse was laid prostrate on the base of the fracture impactor with one hind limb fixed. The proximal end of the femur was positioned under the perspective of the C-arm and marked. The impact weight was set to 500 g at a height of 14 cm, which is adequate to fracture the proximal femur.

It is currently thought that geriatric hip fractures should be treated early with surgery to reduce complications and mortality [20]. Therefore, we performed surgical treatment immediately after confirming by imaging that the fracture model was successfully established. A prophylactic dose of gentamicin (5 mg/kg) was administered intramuscularly. The fractured hip was prepared by hair removal and antisepsis, sterilized with iodophors, and covered with an aseptic sheet. A 0.5 cm surgical incision was made on the lateral side of the proximal femur. The skin and subcutis were cut, and the muscles were bluntly separated to expose the fractured ends of the femur and the greater trochanter. A 1.00 mm Kirschner wire was slowly inserted retrogradely into the proximal medullary cavity of the fractured femur with the proximal Kirschner wire piercing the pyriform sinus of the greater trochanter. The wire was then inserted antegradely into the distal medullary cavity of the fractured femur, during which reduction of the fracture was completed. Afterwards, the exposed portion of the Kirschner wire outside the bone was cut, the wound was flushed with iodophor saline, and the incision was closed with a 3-0 silk suture. Immediately after surgery, 2 ml of sterile physiological saline was administered intravenously for resuscitation. The mouse was placed back into the cage and fed individually. After 6 hours, animals were allowed to eat and drink freely. Buprenorphine (0.1 mg/kg) was given every 10-12 hours for the first 3 days after surgery for pain control. The sham group received only anesthesia and resuscitation.

### 2.3. Bacterial Pneumonia Model

*Pseudomonas aeruginosa* was selected as the bacterial pathogen for this study, as it is one of the most common pathogens associated with hospital-acquired pneumonia [21]. Bacteria were prepared as previously described. Briefly, *P. aeruginosa* (ATCC strain 27853) was grown overnight in trypticase soy broth at 37°C with constant shaking. Bacteria were harvested by centrifugation at 6000 × g. The pellet was washed and resuspended in phosphate-buffered saline to an absorbance of 0.5 at 600 nm (4 × 10^8^ colony-forming units [CFU]/ml). Endotracheal intubation was performed immediately after fracture and surgery, and 40 *μ*l of the 4 × 10^8^ CFU/ml bacterial solution was slowly introduced into the trachea. Mice were kept upright for 1 min to ensure that the solution entered their lungs.

### 2.4. Flow Cytometry Antibodies and Reagents

The following fluorescently labeled antibodies were used for flow cytometry: CD4-PE-Cy5 (cat. #553050; BD Pharmingen, San Diego, CA, USA), CD8-FITC (cat. #553031; BD Pharmingen), and CD279-PE (cat. #12-9985-82; eBioscience, San Diego, CA, USA). PD-1 antagonistic mAb (clone RMP1-14, isotype IgG2*α*, *κ*; BioLegend, San Diego, CA, USA) and its isotype control antibody were used for *in vivo* inhibition studies. Antibodies (200 *μ*g in 200 *μ*l saline) were administered intravenously via the tail vein immediately after surgery.

### 2.5. PD-1 Expression in Splenic Immune Cells

Spleens were harvested from mice 1, 3, or 7 days after fracture surgery or sham surgery. Splenocytes were stained with fluorochrome-conjugated antibodies against CD4^+^ T cells (CD4-FITC; cat. #553729; BD Pharmingen), CD8^+^ T cells (CD8-PE-Cy5; cat. #553034; BD Pharmingen), and PD-1 (CD8-PE-Cy5; cat. # 553034; BD Pharmingen). Flow cytometric analysis (50,000 events/sample) was performed on a FACScan (Becton-Dickinson Biosciences, San Jose, CA, USA), and CellQuest software (BD Biosciences, Franklin Lakes, NJ, USA) was used to analyze the data.

### 2.6. T Cell Apoptosis Assays

Apoptosis was quantified by fluorescent staining using a commercially available antibody against active caspase 3 (Cell Signaling Technology Inc., Beverly, MA, USA) as previously described and by flow cytometry using the APO-BrdU™ TUNEL assay kit (BioVision, San Francisco, CA, USA) following the manufacturer's instructions.

### 2.7. Cytokine Analysis

Whole blood was drawn at the time of euthanasia from animals subjected to hip fracture and surgery. Plasma was collected to determine concentrations of TNF-*α*, interleukin- (IL-) 6, IL-1*β*, and IL-10 using enzyme-linked immunosorbent assays (R&D, Minneapolis, MN, USA) according to the manufacturer's instructions. IFN-*γ* levels were measured in splenocytes by ELISA (R&D, Minneapolis, MN, USA) after culture and stimulation with CD3 and CD28 for 6 h.

### 2.8. Bronchoalveolar Fluid and Blood Bacterial Counts

Mice were deeply anesthetized with isoflurane 24 h after bacterial infection. Following sterile preparation, blood was obtained by direct cardiac puncture, and bronchoalveolar (BAL) fluid samples were obtained by tracheal instillation with 1 ml sterile saline. The collected fluid was diluted in sterile saline and plated onto sheep blood agar plates. Plates were incubated overnight at 37°C, and colony counts were determined after 24 h.

### 2.9. Survival Studies

Mice that underwent hip fracture (Fx) and surgery (Sur) were randomly assigned to receive anti-PD-1 antibody, isotype control antibody (Iso), or saline immediately after hip fracture and surgery. Twenty-four hours later, mice were treated with *P. aeruginosa.* The survival rate was then observed for 14 days.

### 2.10. Statistical Analyses

Data analysis was performed using Prism version 6.0 (GraphPad Software, San Diego, CA, USA). Data are presented as mean ± standard deviation. All studies involving two or more groups were analyzed using one-way analysis of variance (ANOVA) with the Tukey post hoc test. Survival studies were analyzed using the log-rank test. Differences were considered significant at *P* < 0.05.

## 3. Results

### 3.1. Proximal Femur Fracture and Intramedullary Surgery Increases Expression of PD-1 on CD4^+^ and CD8^+^ T Cells

**Figure 1:**
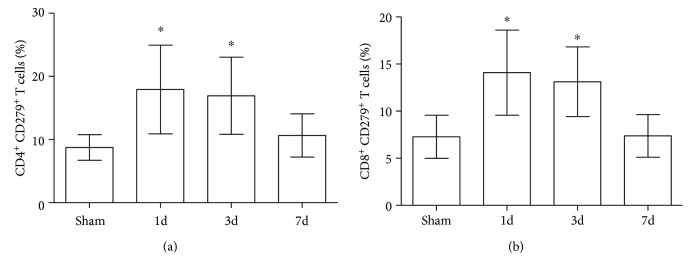
(a, b) PD-1 expression on CD4^+^ and CD8^+^ T cells temporarily increases after hip fracture and surgery. Mice were euthanized 1, 3, and 7 days after hip fracture and surgery or anesthesia alone (sham). PD-1 expression was measured in isolated splenocytes using flow cytometry (*n* = 8 per group). Results are expressed as the mean ± SD. Analysis was performed using one-way ANOVA with the Tukey post hoc test. ^∗^*P* < 0.001 when compared to the sham group.

PD-1 expression on the surface of splenic CD4^+^ and CD8^+^ T cells was found to be significantly increased in mice that underwent intramedullary nailing of hip fractures compared with the sham group 1 and 3 days after surgery (*P* < 0.05). Seven days after surgery, levels decreased to that of the sham group (Figure 1).

### 3.2. Antibody Blockade of PD-1 Inhibits Splenic T Cell Apoptosis

**Figure 2:**
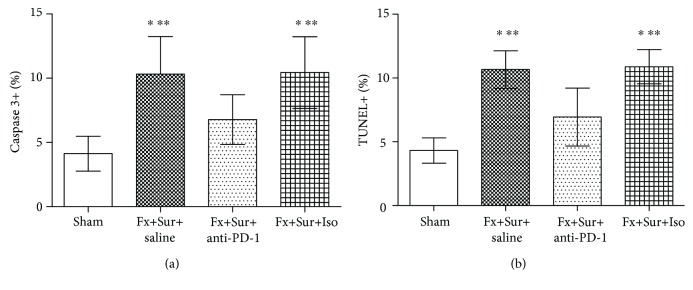
(a, b) Antibody blockade of PD-1 inhibits splenic T cell apoptosis. Immediately following intramedullary nailing of hip fractures (Fx+Sur) or sham surgery (sham), mice were randomly assigned to receive anti-PD-1 antibody (anti-PD-1), isotype control antibody (Iso), or saline. Splenocytes were harvested 24 h after surgery. Apoptosis was quantified by active caspase 3 or TUNEL assays (*n* = 8 per group). Results are expressed as the mean ± SD. Analysis was performed using one-way ANOVA with the Tukey post hoc test. ^∗^*P* < 0.001 when compared to the sham group and ^∗∗^*P* < 0.001 when compared to the Fx+Sur+anti-PD-1 group.

Total splenocytes and CD3^+^ T cell subsets were quantified 48 h after mice that underwent intramedullary nailing of hip fractures or sham surgery were treated with anti-PD-1 antibody, isotype control antibody, or saline. Anti-PD-1 treatment prevented fracture and surgery-induced increases in apoptosis, as detected by active caspase 3 and TUNEL assays (Figure 2).

### 3.3. Antibody Blockade of PD-1 Restores IFN-*γ* Production in Isolated Splenocytes

**Figure 3:**
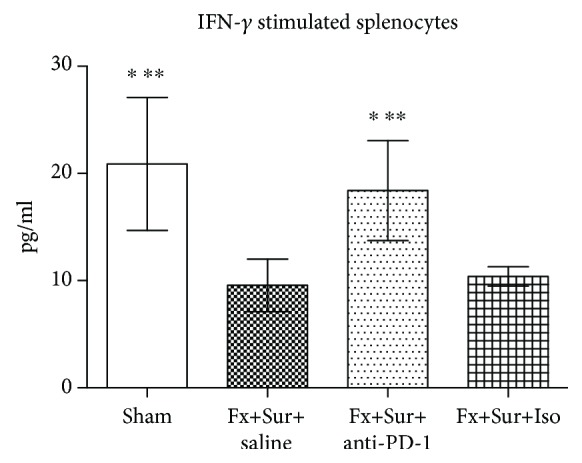
Antibody blockade of PD-1 restores IFN-*γ* production in stimulated splenocytes after hip fracture and surgery (Fx+Sur). Splenocytes were harvested 24 h after surgery. IFN-*γ* levels were determined after stimulation with CD3 and CD28 (*n* = 8 per group). Results are expressed as the mean ± SD. Analysis was performed using one-way ANOVA with the Tukey post hoc test. ^∗^*P* < 0.001 when compared to the Fx+Sur group and ^∗∗^*P* < 0.001 when compared to the Fx+Sur+Iso group.

We evaluated IFN-*γ* production by splenocytes following hip fracture and surgery to determine the ability of the host to mount an effective inflammatory response to a secondary insult. Isolated splenocytes from mice that received anti-PD-1 antibody (Fx+Sur+anti-PD-1 group) produced more IFN-*γ* after stimulation with CD3 and CD28 than splenocytes from mice that received isotype control antibody (Fx+Sur+Iso group) (Figure 3).

### 3.4. Anti-PD-1 Antibody Reduces the Release of Inflammatory Factors after Hip Fracture and Surgery in Aged Mice

**Figure 4:**
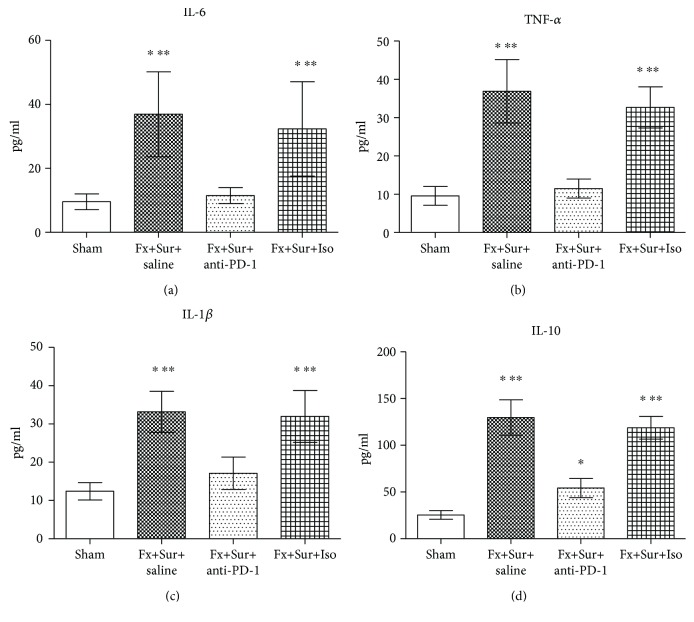
(a–d) Levels of IL-6, TNF-*α*, IL-1*β*, and IL-10 were significantly higher in mice that underwent fracture and surgery (Fx+Sur) than mice that underwent sham surgery. Animals treated with anti-PD-1 after surgery had significantly lower levels of IL-6, TNF-*α*, IL-1*β*, and IL-10 than animals treated with saline or isotype control (Iso) (*n* = 8 per group). Results are expressed as the mean ± SD. Analysis was performed using one-way ANOVA with the Tukey post hoc test. ^∗^*P* < 0.001 when compared to the sham group and ^∗∗^*P* < 0.001 when compared to the Fx+Sur+anti-PD-1 group.

Intramedullary nail fixation of proximal femoral fractures led to systemic inflammation, including IL-6, TNF-*α*, IL-1*β*, and IL-10. However, animals treated with anti-PD-1 after surgery had significantly lower levels of IL-6, TNF-*α*, IL-1*β*, and IL-10 than animals treated with saline and isotype control (Figure 4).

### 3.5. Anti-PD-1 Antibody Improves Survival and Decreases Susceptibility to Bacteria

**Figure 5:**
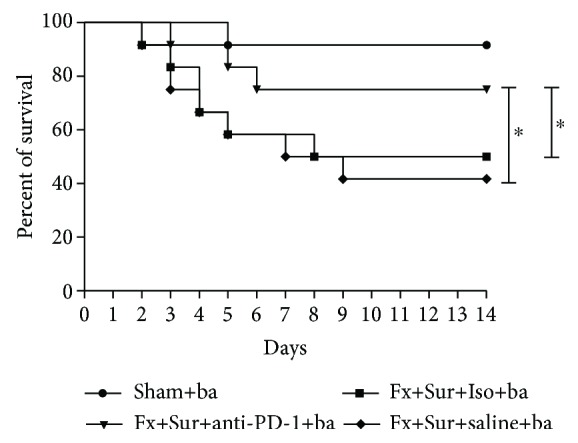
Antibody blockade of PD-1 improves survival after infection. Mice were treated with anti-PD-1 antibody (anti-PD-1), saline, or isotype control antibody (Iso) immediately after hip fracture (Fx) and surgery (Sur). Twenty-four hours later, mice were treated with *P. aeruginosa* (ba). Survival was recorded for 14 days (*n* = 12 per group). Results are expressed as the mean ± SD. Analysis was performed using the log-rank test. ^∗^*P* < 0.001 when compared to the Iso or saline group.

**Figure 6:**
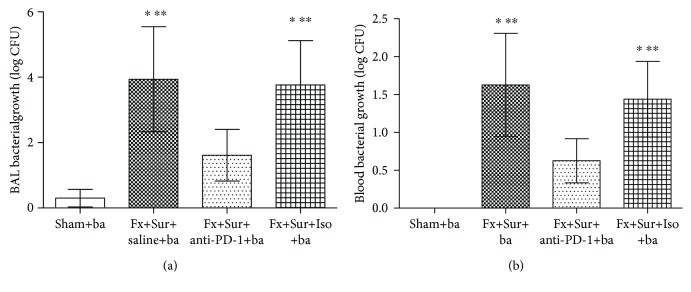
(a, b) Bacterial counts in BAL fluid and blood collected 24 h after *P. aeruginosa* infection (*n* = 8 per group). Results are expressed as the mean ± SD. Analysis was performed using one-way ANOVA with the Tukey post hoc test. ^∗^*P* < 0.001 when compared to the sham group and ^∗∗^*P* < 0.001 when compared to the Fx+Sur+anti-PD-1 group.

When mice exposed to *P. aeruginosa* after hip fracture and surgery were treated with saline or isotype control, their survival rates were significantly lower (41.7% and 50%, respectively) than that of mice exposed to *P. aeruginosa* but given sham surgery (91.7%). However, mice treated with anti-PD-1 antibody had an improved survival rate (75.0%) compared to mice treated with saline (41.7%; *P* < 0.001) or isotype control (50.0%; *P* < 0.001) (Figure 5). Moreover, bacterial counts in both BAL fluid and blood were lower in mice treated with anti-PD-1 antibody than in animals treated with saline or isotype control (Figure 6).

## 4. Discussion

Femoral intertrochanteric fracture is a common injury in the elderly. Currently, closed reduction and intramedullary nail fixation are commonly used for treatment. However, pulmonary infection is a common complication during the perioperative period and significantly affects the therapeutic effects [22]. It is traditionally held that lung infections after hip fracture surgery in the elderly are mainly attributable to long-term bed rest and that most are associated with hypostatic pneumonia [23]. With the development of internal fixation technology for fractures and continuous improvements in nursing quality, elderly patients are able to get up and move around early after surgery, and the amount of recommended bed rest has been greatly reduced. However, rates of lung infection and mortality after hip fracture have not decreased significantly in the past 30 years [24]. This prompted us to further explore the underlying causes of the high incidence of lung infection after hip fracture surgery in the elderly in order to target prevention and treatment strategies.

As the study of posttraumatic immunology has expanded in recent years, it has gradually been revealed that immune suppression caused by trauma and surgery is closely related to the occurrence of complications such as pulmonary infection, sepsis, and multiple organ failure [25, 26]. Our previous animal study confirmed for the first time that hip fracture and intramedullary nail fixation in aged rats can cause significant systemic inflammatory reactions, pathological damage to lung tissue, and increased susceptibility to bacterial lung infections; however, the precise mechanisms involved remained unclear [10]. As an important immunosuppressive factor, PD-1 negatively regulates the immune response [27]. The specific binding of T cell receptor (TCR) and major histocompatibility complex molecule-antigen peptide complexes with costimulatory signals is a key to T cell activation and differentiation. PD-1 is transiently expressed on the surface of activated T cells; however, sustained TCR stimulation and the production of certain cytokines can lead to increased expression of PD-1 on T cells [28]. Binding of PD-1 to its ligand weakens TCR signal transduction through the PI3K/AKT signaling pathway. This inhibits T cell differentiation, proliferation, and cytokine secretion, thereby expediting T cell apoptosis [29]. Many studies have shown that the expression of PD-1 is significantly increased in patients with malignant tumors [30], viral infections [31], sepsis [32], and autoimmune diseases [33]. The blocking of PD-1, or its ligand PD-L1, can lead to positive therapeutic effects. Although it was known that PD-1 plays an important role in the immunosuppressive processes associated with various chronic diseases, the role of PD-1 in trauma-induced immunosuppression was not clear [34]. This study used a model of femoral intertrochanteric fracture and intramedullary nail fixation to assess PD-1 expression and the effect of a PD-1 antagonist on the susceptibility to bacteria and mortality of aged mice.

In this study, we report for the first time that hip fractures and intramedullary nail fixation significantly increases the expression of PD-1 in T cells of aged mice. PD-1 expression peaked 1 day after surgery, then gradually decreased until reaching levels similar to that of the sham group 7 days after surgery. Furthermore, anti-PD-1 significantly reduced T cell apoptosis in the spleen. IFN-*γ* is an inflammatory cytokine with important roles in fighting infection. Since T cells play a central role in the elimination of bacteria from the body and IFN-*γ* is secreted mainly by T cells, levels of IFN-*γ* reflect, to a degree, T cell functioning and the ability of the host to resist infection [35]. In this study, the secretion of IFN-*γ* by T cells was significantly reduced following hip fracture in aged mice, whereas anti-PD-1 antibody significantly increased the secretion of IFN-*γ*, thereby restoring to a certain extent T cell function.

It is known that trauma and surgery can lead to immune/inflammatory disorders involving increased expression of various proinflammatory and anti-inflammatory factors such as IL-6, TNF-*α*, and IL-10 [36, 37]. Furthermore, the severity of immune/inflammatory disorders is closely related to the occurrence of infectious complications [38, 39]. In this study, we found that anti-PD-1 can reverse the overexpression of inflammatory factors and reduce the severity of immune/inflammatory disorders caused by fracture and surgery. Because anti-PD-1 can reduce T cell apoptosis, partially restore T cell function, and regulate posttraumatic immune and inflammatory responses, we hypothesized that antibody blockage of PD-1 may reduce susceptibility to bacteria and improve prognosis. To test this, we established a model of *P. aeruginosa* pneumonia by intratracheal instillation of *P. aeruginosa* bacteria after fracture and assessed the effect of anti-PD-1 intervention. The results showed that anti-PD-1 significantly increased bacterial clearance rates and the 14-day survival rate of mice from 41.7% to 75%.

Pulmonary infection is an important contributor to the high mortality rates associated with hip fractures. These infections are difficult to cure because antibiotics are currently the only available drugs to treat bacterial infections. Therefore, improving immune functioning is vital to enhancing bacterial elimination. However, at present, few orthopedic surgeons are concerned by changes in the immune function of elderly individuals undergoing surgery to treat fractures. Our study showed that hip fracture and surgery can cause significant immune/inflammatory disorders in the elderly, including excessive secretion of inflammatory cytokines, high expression of the immunosuppressive molecule PD-1, and increased apoptosis of T cells, leading to increased susceptibility to pulmonary infection and death. Furthermore, this study suggests that the incidence of pulmonary infections after hip fracture in the elderly may be more closely related to posttraumatic immune/inflammatory disorders than to long-term bed rest. However, administration of PD-1 antibody can significantly reduce inflammation, partially restore T cell function, enhance bacterial clearance, and increase the 14-day survival rate after lung infection. In addition to treating fractures, orthopedists must be aware of the systemic effects of fractures, surgical trauma, blood loss, pain, and anesthesia on the elderly. During the treatment of hip fractures, concepts of “damage control” and “enhanced recovery after surgery” should always be adopted to protect the overall well-being of the elderly [40, 41].

Our findings are based on animal experiments that cannot fully replicate the postoperative clinical immune status of elderly patients with hip fractures, because the elderly patients could be complicated by many comorbidities (diabetes, cardiovascular or respiratory diseases, neoplasm, immune diseases, etc.) which affect the immune system and survival. Therefore, in follow-up work, we will monitor PD-1 in perioperative peripheral blood of clinical patients and determine whether PD-1 is correlated with infectious complications. Moreover, the specific molecular mechanism underlying PD-1 overexpression after hip fracture and surgery still requires further study.

In conclusion, this study demonstrated that hip fracture and surgery in elderly mice can cause significant immune/inflammatory disorders associated with increased PD-1 expression and that antibody blockade of PD-1 can partially restore T cell function, decrease susceptibility to bacteria, and reduce mortality.

## Data Availability

The date used to support the findings of this study are available from the corresponding author upon request.
